# Who Do I (Not) Ask to Play my Lottery? Effects of Perceived Positive and Negative Agency, Communion and Luck on the Illusion of Control by Proxy

**DOI:** 10.1007/s10899-023-10228-9

**Published:** 2023-06-20

**Authors:** Mateusz Polak, Katarzyna Stasiuk, Karolina Chodzyńska

**Affiliations:** Institute of Applied Psychology, Faculty of Management and Social Communication, Jagiellonian University, ul. Łojasiewicza 4, Krakow, 30-348 Poland

**Keywords:** Illusion of control, Agency, Communion, Luck, Lottery

## Abstract

The paper investigates the illusion of control by proxy in games of chance - an attempt to exert control by assigning it to others who are perceived as more capable, communable or luckier. Following up on research by Wohl & Enzle, who showed participants’ preference to ask lucky others to play a lottery instead of doing it themselves, we included proxies with positive and negative qualities in the domains of agency and communion, as well good and bad luck. In three experiments (total N = 249) we tested participants’ choices between these proxies and a random number generator in a task consisting of obtaining lottery numbers. We obtained consistent preventative illusions of control (i.e. avoidance of proxies with strictly negative qualities, as well as proxies with positive communion but negative agency), however we observed indifference between proxies with positive qualities and random number generators.

When gambling, agents seek control over random chance. This illusory control can be derived from perceived ‘skill’ components of a gamble (*I need to press the button hard to win the jackpot*), from personal luck (*Today’s my lucky day to win the lottery*), but also from ‘using’ other people’s luck or skill for the agent’s own gain. Asking a person who is perceived as lucky to spin the roulette in our stead is an example of such *illusion of control by proxy*, which is defined as *an attempt to exert control by assigning control to more capable (luckier) others to act for [an agent]* (Wohl & Enzle, 2009). This paper investigates whether the illusion of control by proxy can be observed for proxies perceived as lucky, but also for proxies perceived as competent (high agency) and/or moral (high communion). On the other side, we investigate whether proxies with negative qualities (unlucky, incompetent, immoral) cause an opposite effect, with agents avoiding them.

## Illusion of Control

Illusion of control is one of many cognitive biases which let us satisfy our epistemic needs and find some desired certainty in unpredictable, random situations. In her original research, Ellen Langer defined illusion of control as *an expectancy of personal success inappropriately higher than the objective probability would warrant* (Langer, 1975, p.313). According to Langer’s idea, illusion of control manifests itself by individuals perceiving purely chance situations as skill-related, and applying these ‘skills’ in order to control these situations. However, as Goodie et al. (2019) point out, there is an inconsistency as to the exact definition of illusion of control. In the context of gambling, illusion of control is understood as a skill orientation toward gambling (Joukhador, MacCallum & Blaszczynski, 2003), beliefs about skills and strategies to control games of chance (Steenbergh, Meyers & May, 2002), rituals and behaviors to increase chances of winning (Raylu & Oei, 2004) or constructs encompassing actual attempts to change the situation and attempts to influence a ‘higher power’ to grant a favorable outcome (Ejova, Delfabbro & Navarro, 2015). Moore and Ohtsuka (1998, p. 341) defined the illusion of control as “*the invocation of superstitious behavior as a flawed way of attempting to influence winning or losing at gambling”*. Toneatto (1999, p. 1594) defined it as “*a tendency to believe that there is a greater probability of obtaining a chance-determined outcome than would be dictated solely by random chance”*.

The above definitions vary in the way that the underlying mechanisms of illusion of control are perceived. The ‘classical’ approach (cf. Langer, 1975; Langer, 1983) would be to assume that people search for elements of skill in chance situations, and then use the skill to influence these situations. Most of the above definitions, however, lead to the conclusion that illusion of control may be present whenever an individual believes they have a better chance of winning than the objective probability would state. The reason for this belief may be because the individual sees the situation as skill-dependent (e.g. dice can be manipulated by skillfully throwing them, or a slot machine can be manipulated by pressing the buttons with specificspeed or force, Henslin, 1967; Griffiths, 1999). It may also be that the person believes in superstitions which are expected to increase the chances of a favorable outcome (e.g. carrying lucky charms, blowing on dice or performing other rituals which are not considered ‘skill’ but are expected to increase luck). There is ample evidence regarding gambling superstitions and their association with perceived luck (e.g. Wiseman and Watt, 2004; Darke and Freedman, 1997; Torgler, 2007; Griffiths and Bingham, 2005).

Luck in the context of illusory control was investigated by Wohl and Enzle (2002, 2003). Specifically, following the differentiation by Keren and Wagenaar (1988), they introduced the term *personal luck* as opposed to chance events. Personal luck is considered a stable quality possessed by a person – it can be thought of as a (perceived) deployable skill which can be used to exert control over situations which are in fact random in nature. Wohl and Enzle consider personal luck to be a manifestation of *sympathetic magical thinking* (Nemeroff & Rozin, 2000): when control over an outcome is important (i.e. there is a need for control), people may consider causal forces which do not exist within the world of known physical laws – in other words, they decide to believe in the metaphysical, because it gives them control. There are specific situational characteristics which allow individuals to apply their personal luck. One of these characteristics is choice. If a person can choose a lottery ticket themselves, they have an opportunity to ‘use’ their luck to pick the winning ticket. If, on the other hand, the ticket is given to them by another person, people may believe that the other person’s luck (and not theirs) is responsible for the outcome. Similarly, if a gambler pushes the buttons of a slot machine, it gives them an opportunity to exercise their luck, but if they ask someone else to do it, that someone else’s luck will influence the outcome. It seems that we believe luck to be physically related to the person who takes the action, just as skill is physically related to the person using that skill.

Anecdotal situations describing gamblers trying to exercise personal luck are plenty. According to Wohl and Enzle (2002), there is a desire to handle (have physical contact with) objects which are central to the game of chance, when deployment of luck is most relevant –showing the need for ‘physical connection’ between the person exercising their personal luck and the object on which the luck needs to be deployed. This can be observed in gamblers clutching their lottery tickets when results are presented, and in superstitious roulette players tightly holding their ‘lucky charms’ when the wheel spins. There are, however, situations when the opposite is true. There are numerous movie scenes taking place in casinos, in which the protagonist will ask someone (e.g. their significant other) to blow on the dice, kiss it, or otherwise have contact with it before throwing them – or to throw the dice in the protagonist’s stead (examples of such movies are Indecent Proposal (1993), Diamonds are Forever (1971), or even Iron Man (2008). The idea is that the protagonist ‘borrows’ luck from the other person to get the win. Wohl and Enzle (2009) investigated such a situation – giving control of our own game of chance to another person who is perceived as lucky – and called it the *illusion of control by proxy*.

## Illusion of Control by Proxy

According to their definition, *illusion of control by proxy* is *an attempt to exert control by assigning control to more capable (luckier) others to act for them* (op. cit., p. 184). Illusion of control by proxy is therefore aimed at ‘using’ another person’s luck in order to gain a favorable outcome for oneself, and is consistent with the general definition of illusion of control, albeit using a different control mechanism than one’s own skill or luck. In a series of three experiments, Wohl and Enzle demonstrated the presence of illusion of control by proxy in a Canadian population. In a series of three experiments, Wohl and Enzle paired participants with a confederate (seemingly another participant). This confederate either presented themselves as lucky or neutral. In Experiment 1, the lucky confederate stated that they had an opportunity to touch the Hockey Hall of Fame Lucky Loonie (a coin which gave luck to the Canadian hockey team), and that *ever since they [touched] it, they can’t lose (…) they won [at gambling] every time since they touched that coin* (p. 186). The neutral proxy simply said that they did not touch the coin. In Experiments 2 and 3, the lucky confederate stated that *he thinks he is lucky as he always tends to win, regardless of the gambling game played*, and the neutral proxy stated that *he often goes to the casino and plays the lottery*. In each of the three experiments, the true participants then had the opportunity to either take a gamble themselves (i.e. choose a lottery ticket or spin a virtual roulette), or to ask the confederate (proxy) to do it for them. Regardless of this choice, the true participants would receive any potential winnings. It turned out that in all three experiments, participants gave up control to the lucky proxy very often, while few participants asked the neutral proxy to gamble for them.

While the studies by Wohl and Enzle (2009) consistently demonstrate the *illusion of control by proxy*, there are a few issues worth discussing. Firstly, the way the confederates described their luck to the participants was very strong. In all experiments the confederates stated that they *always win* and/or *cannot lose*. Clearly this description is not very ecologically valid and does not seem believable. More importantly, such a description may have lead participants to give control to the proxy out of pure curiosity – to test the statement that the confederate cannot lose. Firstly, participants may want to test this statement because it may be true – it is a psychological experiment after all, so the game may have been rigged for the other participant to always win. Moreover, participants may get satisfaction (out of a self-esteem boost or tending to their cognitive motivation) from finding out that the other participant was overconfident (or outright dishonest), and contrary to their statement does not always win, making it a case of *you win, I get the money; you lose, I get satisfaction*. Therefore in the current research we decided to make the descriptions of proxies more believable, to check whether the illusion of control by proxy will still be present in such a case.

More importantly, as stated by Wohl and Enzle (2009) they did not test an unlucky-proxy condition, which would be a natural continuation of the research. The current paper investigates effects of offering the choice of an unlucky proxy to the participants.

Investigating proxies with negative traits (undesirable ones) should, in theory, show that participants prefer to act themselves rather than choose such a proxy. Otherwise there is no point in letting an unlucky person decide in our stead. The issue of bad luck is however important when we consider the promotion and prevention regulatory focus (Higgins, 1997, 1998). Langens (2007) implemented the regulatory focus theory to the illusion of control, stating that illusion of control is most likely to occur when an individual is focused on promotion, as promotional focus causes a sensitivity to matches to the desired state (i.e. wins), which together with the actions which led to the desired outcome, generate ‘double positives’ typical for confirmation bias (Bruner, Goodnow & Austin, 1956; Nickerson, 1998). While a lucky proxy should activate promotional focus (winning), an unlucky proxy could activate preventative focus (not losing). In his Study 2, Langens (2007) manipulated promotion/prevention focus by making participants play a game focused either around winning or around seeking security. It turned out that a promotion focus resulted in a higher perceived control and a greater amount of reported ‘strategies’ to manipulate a subsequently played game of chance – essentially showing that promotion focus is associated with a higher illusion of control. If we assume that a lucky proxy facilitates focusing on winning, and an unlucky proxy makes participants think about a way to avoid losing, then on the one hand an unlucky-proxy condition may reduce the illusion of control, and on the other hand the unlucky proxy does not conflict with the participants’ own perceived luck, which could create a stronger preference for own control while at the same time reducing perceived chances of winning.

## Postulated Effects of Agency and Communion on the Illusion of Control by Proxy

Since the illusion of control is associated with skill (personal luck being a particular type of skill), it is possible that other qualities presented by proxies could create the illusion of control by proxy in situations of random chance. We hypothesized that these qualities may refer to agency and communion - two fundamental dimensions underlying social judgements.

Agency and communion represent broad clusters of behavior, accounting for 82% of variance in perceptions of everyday social behaviors, and broad categories of social cognition which exist in the minds of human observers. Whereas communion relates to functioning in social relations and involves such qualities as morality, loyalty and helpfulness, agency reflects goal achievement and task functioning, and comprises attributes such as competence, efficiency and decisiveness (Fiske, Cuddy & Glick, 2006).

Although both communion and agency are fundamental in social perception, substantial evidence suggests that communion is primary – it is preferentially processed at early stages of information processing and carries higher weights in affective and behavioral reactions (Abele & Bruckmiller, 2011). This is because communion qualities are other-profitable, i.e. directly beneficial for other people when positive, and directly harmful for them when negative. Agency qualities, on the other hand, tend to be self-profitable, because they directly and unconditionally affect an agent’s chance of achieving personal goals (Abele & Wojciszke, 2007, 2014; Peeters, 2001). However, agency may also contain a subset of other-profitable attributes. This may be the case when another person is efficient (or perceived as efficient) in the pursuit of goals which are desirable for an individual. In many areas of functioning, people feel unable to control some circumstances which affect their everyday lives. In such situations they usually strive to obtain outcomes consistent with their well – being and values through the exercise of *proxy agency* (Bandura, 2001). An example is when people seek advice from professional advisors because they feel they do not have enough knowledge and competence to take the appropriate action or to choose the better option. Sometimes they give up control because they ascribe experts some ‘magical traits’ such as being able to predict the future. Belief in such ‘magical traits’ is reflected in some people’s assumption that financial advisors are able to time the market because they are able to foresee when the market will plunge or soar ahead of time – effectively predicting the future, as such is the naïve understanding of how experts are able to obtain better returns from investments than laymen. Taking the above into account, and also following the assumption that people may perceive chance situations as skill related, we hypothesized that in the context of lottery, (which is associated with the categories of money and finance), people may tend to use proxies who are ascribed high competence in finance or economics.

In the current research, we also assumed that people may be willing to transfer control not only when a proxy is skillful and competent in a relevant domain (here: finance), but also when a proxy is ascribed high morality, and because of that “deserves” the positive outcome (i.e. to win the lottery). This assumption stems from the *just world theory*, which states that people need to believe in a just world in which everyone gets what they deserve and deserves what they get (Lerner, 1980). This belief serves important adaptive functions because it enables agents to deal with their social environment as though it was predictable and manageable. A central aspect of the theory is that people believe that there is an appropriate relation between the value of people and the value of their outcomes. To preserve the belief in just world people employ various cognitive and behavioral strategies, such as immanent justice reasoning explanations of events (for a review see Callan, Sutton, Harvey & Dawtry, 2014). Such explanations link observed negative outcomes with prior immoral behaviors (even if there is no rationale for such a causality), as well as positive outcomes which are attributed to morally positive but objectively unrelated behavior. In one of the studies on immanent justice reasoning participants thought that a lottery windfall was a consequence of a man’s prior moral behavior to a greater extent when he was presented as a good and kind person (Callan, Ellard & Nicole, 2006).

Following these results, we assumed that the *just world theory* is not just used to explain prior events, but also as a basis for expectations that good (bad) things will happen to good (bad) people, which may be reflected in the illusion of control by proxy. Specifically, we hypothesized that people will be more willing to give up control in situations of random chance to proxies ascribed positive moral behavior or traits, who ‘deserve’ the positive outcomes more than an immoral proxy.

To sum up, the main goal of our research was to further investigate the *illusion of control by proxy* in its ‘positive’ and ‘negative’ forms (i.e. giving control to proxies with positive qualities such as personal luck, vs. avoiding giving control to proxies with negative qualities). We wanted to replicate results by Wohl and Enzle (2009) with proxy descriptions which would be more believable, to test whether their results may have been caused by curiosity rather than illusion of control. We also wanted to investigate the effects of an unlucky proxy and whether such a proxy would be actively avoided by participants, which would also be an indication of illusion of control. Furthermore, we wanted to investigate whether the illusion of control by proxy goes beyond the trait of personal luck, i.e. whether the agency and communion of a proxy can cause a similar effect, and whether agency or communion is the more preferred trait for a proxy in the context of random chance games.

To test our predictions, we designed three experiments using the systematic replication and modification rule. In all experiments, the participants were asked to draw three numbers to take part in a lottery, but they could not do it personally. Instead, they could ask another person (proxy) to choose for them, or use a random number generator (RNG). In all experiments the respondents could choose among the proxies that were described by positive or negative behaviors (Experiment 1 and Experiment 2) or traits (Experiment 3) from the agency and communion dimensions, or with positive or negative luck in games of chance. The data and study materials (the used scenarios) are available at OSF https://osf.io/gsyjr/?view_only=f42adf2240284851a16fb02a4b79ce0a.

## Experiment 1

Experiment 1 was aimed at measuring the participants’ preferences between proxies varying in their agency, communion and luck, who would provide lottery numbers for the participants. In line with the comments by Wohl and Enzle (2009), who stated that direct social contact between the participant and the proxy may generate unwanted pressure, we decided to use a computerized procedure. We also wanted to include both positive and negative traits (i.e. low agency, low communion and bad luck), so we provided participants with the option to use a random number generator (RNG) instead of the proxy. Should neither proxy have desirable qualities for the participants, they would be able to get completely random numbers from a machine. However, we did not give participants the option to select lottery numbers themselves, as this could create a strong conflict between their own illusory control and the illusion of control by proxy. This stands in opposition to the research by Wohl and Enzle (2009), who gave participants the choice between their own illusory control (choosing their lottery ticket) and illusory control by proxy. This change was also made to provide clearer results regarding the undesirable proxies – when faced with a choice between choosing themselves or asking an unlucky proxy to choose, participants’ preference toward choosing themselves could be explained either by a negative illusion of control by proxy (i.e. avoidance of unlucky proxy) or by ‘standard’ illusion of control (i.e. exercising own ‘skill’/personal luck). RNG was implemented as a baseline choice with as little room for illusory control as possible.

In three experiments we wanted to investigate whether a proxy with high agency and low communion (consistent with the ‘skill’ aspect of the illusion of control) is preferred to a proxy with low agency and high communion (consistent with the ‘just world’ expectations). We also wanted to investigate whether high agency and communion is preferred to good luck (actual skill vs. personal luck trait). Finally, we wanted to check whether bad luck or low agency and low communion is perceived as more undesirable. The data are available at https://osf.io/r4pbv/?view_only=None.

### Participants

We recruited a total of 107 students (74 females and 33 males) aged 18–40 years (M = 21.83, SD = 3.98) on campus to participate in the study. Participants were approached by a research assistant in the hall of the Institute of Applied Psychology at the Jagiellonian University in Krakow (Poland). They were asked to take part in the computer experiment on decision-making in lottery games. They didn’t receive payment or credit for participation, informed consent was obtained from all participants. We assumed that the effect size would be at least moderate (~ 0.3), based on the results by Wohl and Enzle (2009) who obtained rather large effects. Assuming α = 0.05 and df = 2, the sample size of 107 offered a statistical power of 0.8. The study protocols (for three experiments) was reviewed and approved by the Ethics Committee of the Institute of Applied Psychology, Jagiellonian University in Krakow. The questionnaire collected no identifying personal data from the participants.

### Materials and Procedure

The study was conducted using a computerized procedure programmed in PsychoPy (Peirce et al., 2019). Participants were given the following instructions: *You will take part in an experiment about decision-making. You need to pick three numbers which will take part in a lottery. However, you cannot pick them yourself. Instead, you will be presented with descriptions of people whom we asked to choose numbers which they think would win the lottery. On each screen, you will be able to select the person you would like to give you the number (a financial advisor or a lottery player), or to use a random number generator (RNG) instead. Based on your decisions, you will receive three numbers between 1 and 100. After the experiment, one number between 1 and 100 will be drawn, and if any of your three numbers matches this number, you will receive 10 PLN*^1^. *Information about financial advisors was obtained from their customers; information about lottery players was obtained from themselves.*

Following this instruction, participants were presented with three on-screen simultaneous descriptions of two proxies (and RNG) at a time. Proxies were either financial advisors (as we wanted to clearly link their agency and communion behaviors to the context of money) or lottery players (carrying good or bad personal luck). These proxies were given a short description representing their agency and communion actions (e.g. the high agency, low communion proxy was described as ‘*He knew the differences between various investment offers. He tried to convince me to additional investments I did not need.’*). The lottery player proxies were described as lucky or unlucky, e.g. ‘*He played the national lottery regularly for several years. He got the second prize three times and a small prize seven times’*. These descriptions were based on a pilot study (N = 90) to select ones with the desired agency, communion and luck levels. Decision options were presented in the following sets: (1) High Agency, Low Communion advisor vs. Low Agency, High Communion advisor vs. RNG, (2) High Agency, High Communion advisor vs. Lucky lottery player vs. RNG, and (3) Low Agency, Low Communion advisor vs. Unlucky lottery player vs. RNG. The order in which proxies within each set were presented was changed randomly (RNG was always last). The order of decision tasks was fixed. Participants could select either the first proxy in a set by pressing the left arrow key, the second proxy by pressing the right arrow key, or RNG by pressing space. Following the procedure, three random numbers were provided to the participants (regardless of their decisions). A questionnaire measuring the perceived agency, communion and luck of each proxy was then presented to the participants, to check whether the descriptions accurately manipulated these traits, followed by selecting the winning number.

### Results

The tasks presented to participants in our research were simple discrete choice experiments (DCEs’; Louviere et al., 2000). To analyze the main effects, we used the traditional approach of testing for preferences (for example see Lichtenstein and Slovic, 1971; Kahneman and Tversky, 1979) which assumes that if all options are equally valued by the agents, the distribution of preferences is not different from random chance (i.e. there are no significant intersubjective preferences). For the three decision-making tasks, we therefore compared frequencies of choices to random chance using chi-square tests. Effect sizes calculated using Cramer’s V were considered negligible below 0.10, weak between 0.10 and 0.20, moderate between 0.20 and 0.40 and relatively strong between 0.40 and 0.60 (we did not obtain higher values; cf. Lee, 2016 for an interpretation). In the first task, 43 out of the 107 participants (40%) chose the High Agency-Low Communion proxy, 36 participants (34%) chose RNG, and 28 participants (26%) chose the Low Agency-High Communion proxy. These differences were weak (χ^2^(2,N = 107) = 3.159, Cramer’s V = 0.12). In the second task, 40 participants (37%) chose the Lucky proxy, 35 participants (33%) chose RNG and 32 participants (30%) chose the High Agency-High Communion proxy. Again, these differences were negligible (χ^2^(2,N = 107) = 0.916, Cramer’s V = 0.06). In the third task, 68 participants (64%) chose RNG, 27 participants (25%) chose the Unlucky proxy, while only 12 participants (11%) chose the Low Agency-Low Communion proxy. The effect size was relatively strong (χ^2^(2,N = 107) = 47.121, Cramer’s V = 0.47), revealing a strong undesirability of both the unlucky proxy and the low agency-low communion one.

As a general measure of illusion of control by proxy, we also tested whether proxies with positive traits were selected more often than RNG (tasks 1 and 2). As already noted, in task 3 proxies were selected less often than RNG because they both carried negative traits (37 vs.70, χ^2^(1,N = 107) = 10.178, Cramer’s V = 0.30). In Task 1, proxies were chosen by 72 participants and RNG was chosen by 35 participants (χ^2^(1,N = 107) = 12.794, Cramer’s V = 0.35). In Task 2, proxies were chosen by 74, and RNG by 33 participants (χ^2^(1,N = 107) = 15.710, Cramer’s V = 0.38). Please note that these results assume that we consider the two proxies as one category and test them against a 50/50 random chance vs. RNG. If Proxy 1 and Proxy 2 are to be considered two separate categories out of three, therefore carrying a 2/3 random chance of being selected, the differences in Task 1 and Task 2 disappear.

**Fig. 1:**
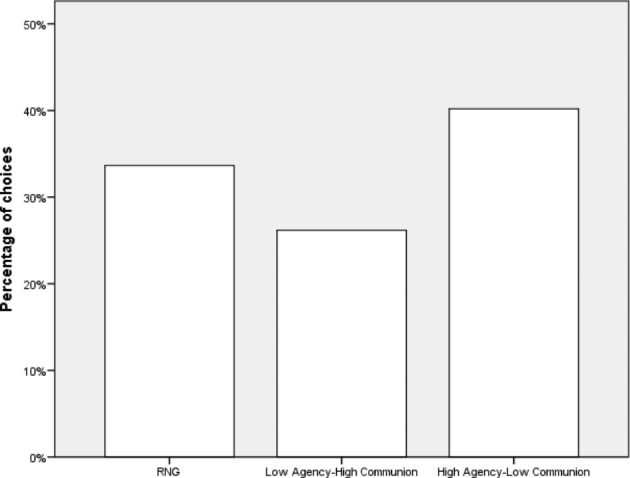
Preferences in Task 1, Experiment 1

**Fig. 2:**
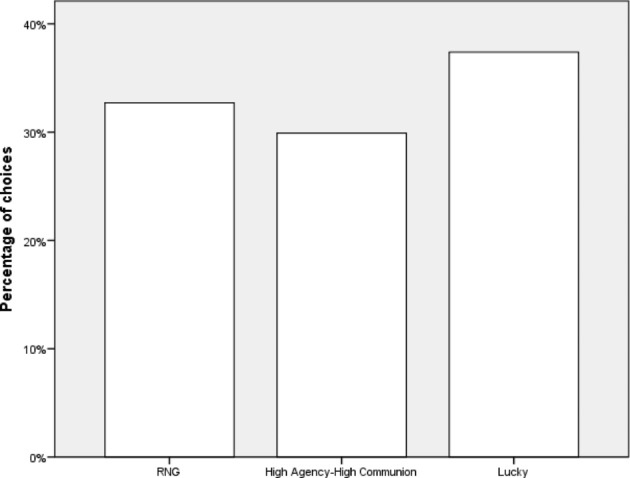
Preferences in Task 2, Experiment 1

**Fig. 3:**
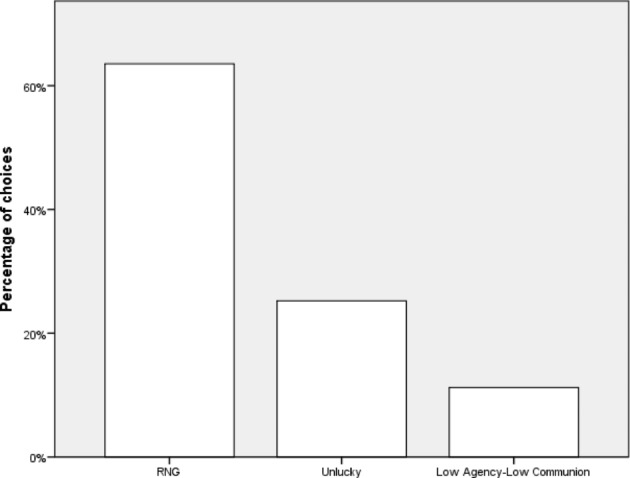
Preferences in Task 3, Experiment 1

Among the participants who chose proxies in Task 1, there was a moderate preference for High Agency-Low Communion (N = 43) over Low Agency-High Communion (N = 28, χ^2^(1,N = 72) = 4.50, Cramer’s V = 0.25). In Task 2, there was a weak preference of the Lucky proxy over the High Agency-High Communion proxy (40 vs. 32, χ^2^(1,N = 72) = 0.865,, Cramer’s V = 0.11). In Task 3, there was a moderate preference of the Unlucky proxy (N = 25) over the Low Agency-Low Communion one (N = 12; χ^2^(1,N = 37) = 4.588, p = .033, Cramer’s V = 0.35). Results are presented in Figs. 1, 2 and 3.

The post-experimental questionnaire showed that the qualities of proxies perceived by the participants were consistent with the descriptions based on the pilot study. Perceived qualities of proxies with examples of descriptions are presented in Table 1.

**Table 1:** Examples of descriptions of proxies (Experiments 1 and 2) and their perceived qualities (Experiment 1)

Type	Description	Agency (− 3;3)	Communion (− 3;3)	Luck (− 3;3)
HA/LC	*He knew the differences between various investment offers. He tried to convince me to additional investments i did not need.*	1.70	− 0.55	0.6
LA/HC	*He did not know the financial situation of companies whose investment products he recommended;* *He informed me about all hidden costs of the investment*	− 1.17	0.98	0.17
HA/HC	*He knew the current stock market trends; He informed me about the size of his cut received from the investment companies.*	2.10	1.94	0.54
LA/LC	*He had little knowledge about the current stock market situation; He recommended aggressive investment even though he knew I could suffer losses.*	− 1.86	-1.64	− 0.16
Good luck	He played the national lottery regularly for several years. He got the second prize* three times and a small prize** seven times. [Comment: *est. value $1200; ** est. value $25]	0.23	0.46	2.10
Bad luck	*He played the national lottery regularly for several years. He did not even win a small prize.*	− 0.36	0.16	− 2.08

Note: HA = High Agency, LA = Low Agency, HC = High Communion, LC = Low Communion

### Discussion

Results of Experiment 1 indicated that there was a strong reverse illusion of control in case of proxies with strictly negative qualities (low agency-low communion proxy and unlucky proxy). Participants preferred RNG over these proxies in most of the cases. This could be considered an illusion of control in the domain of prevention – the participants avoid proxies with qualities which may yield an unfavorable outcome, even though in case of agency and communion these qualities are not rationally associated with the potential result of a random chance game. Moreover this adds to the research by Wohl and Enzle (2009) indicating that a perceived unluckiness of a proxy causes agents to avoid the proxy, just as their research indicated that a perceived luckiness caused agents to seek the proxy’s support in winning the lottery.

Among participants who decided to obtain the lottery numbers from proxies, there is a preference of high agency-low communion over high communion-low agency, and a complementary preference of bad luck over low agency-low communion, indicating that agency may be the most important factor out of the three. This is in line with Langer’s understanding of illusion of control – participants perceive the outcome of a random chance game as skill-dependent, and carry over the proxies’ agency as financial advisors into the domain of lotteries, i.e. the participants seem to assume that a good financial advisor is also more ‘skilled’ at winning lotteries. On the other hand, results did not support the ‘just world’ hypothesis, as low communion was ignored in favor of high agency and vice versa. This indicates that the belief that good people deserve to win lotteries is at least not as important as the belief in skill dependence.

The design used in Experiment 1 (i.e. choices between two proxies and RNG in each task) has a certain methodological-statistical flaw, which prevented us from obtaining clear results in Tasks 1 and 2. In these tasks, most of the participants chose proxies and not RNG, which could potentially be indicative of an illusion of control by proxy. However, as there were two proxies and one RNG, the higher number of proxies selected over RNG may also be the result of random chance. For instance, we can imagine that with two attractive proxy options, participants would prefer proxy over RNG in 66%of the cases, and then would be more or less indifferent between these proxies – this would result in a 33% frequency in all categories. The same would be the result of participants being indifferent between the two proxies and RNG, which is consistent with rational choice, and not indicative of illusion of control by proxy. Therefore we decided to rectify this issue in Experiment 2 by implementing choices between one proxy and RNG at a time.

## Experiment 2

The aim of Experiment 2 was to replicate the results of Experiment 1 using simpler decision tasks, consisting of choosing between one proxy vs. RNG. The main issue was to test whether a general illusion of control by proxy would be present for proxies with positive qualities (high agency, high communion and/or luck) with choices restricted to two options. We also wanted to replicate the findings of Experiment 1 about proxies with negative qualities.

### Participants

We recruited a total of 68 students (50 females and 18 males) aged 18–24 years (M = 21.4, SD = 1.53) on campus to participate in the study (the recruitment procedure was the same as in the Experiment 1)They didn’t receive a payment for participation, informed consent was obtained from all participants .

The sample size was smaller than in Experiment 1, since we only conducted analyses with *df* = 1.

### Materials and Procedure

The experiment was conducted using the same materials and procedure as Experiment 1. The only difference was that decisions were made between one proxy and RNG at a time, rather than between two proxies and RNG. To keep the procedure as close to the previous one as possible, we used three decision tasks (and three lottery numbers) per participant. For half of the participants, the order of the tasks was (1) High Agency-High Communion vs. RNG, (2) Low Agency-Low Communion vs. RNG, (3) Lucky vs. RNG. For the other half it was (1)High Agency-Low Communion vs. RNG, (2) Low Agency-High Communion vs. RNG, (3) Unlucky vs. RNG. The order in which the proxies were presented was randomized. Participants could select the proxy by pressing the Up arrow key, and select RNG by pressing Space.

### Results

**Fig. 4:**
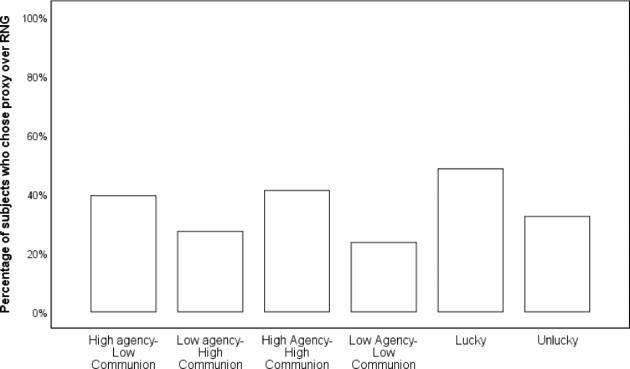
Percentages of participants who chose proxy over RNG (Experiment 2)

Participants’ choices were compared to 50% random chance by means of chi-square tests. It turned out that participants had moderate preferences of RNG over a High Agency-Low Communion proxy (20 vs. 13, χ^2^(1,N = 33) = 1.485, Cramer’s V = 0.21), weak preferences of RNG over a High Agency-High Communion proxy (20 vs. 14, χ^2^(1,N = 34) = 1.059, Cramer’s V = 0.18) and, rather surprisingly, negligible preferences between a lucky proxy and RNG (16 vs. 17, χ^2^(1,N = 34) = 0.030, Cramer’s V = 0.03). There was a relatively strong preference of RNG over a Low Agency-High Communion proxy (24 vs. 9, χ^2^(1,N = 33) = 6.818, Cramer’s V = 0.45), a relatively strong preference of RNG over a Low Agency-Low Communion proxy (26 vs. 8, χ^2^(1,N = 34) = 9.529, Cramer’s V = 0.53) and a moderate preference of RNG over an unlucky proxy (23 vs. 11, χ^2^(1,N = 34) = 4.235, Cramer’s V = 0.35). Results are presented in Fig. 4.

Figure 4. Percentages of participants who chose proxy over RNG (Experiment 2).

Post-experimental questionnaires showed that the perceived qualities of proxies were consistent with their descriptions apart from one case: the Low Agency-High Communion proxy was not perceived as bearing a significantly high communion (M = 0.29, SD = 2.2). Therefore the Low Agency-High Communion proxy can be considered a Low Agency-Neutral Communion one. This seems to be an experimental artifact, as the same proxy with the same description was considered Low Agency, High Communion in Experiment 1. These results are presented in Table 2.

**Table 2:** Perceived qualities of proxies (Experiment 2)

Type	Agency (− 3;3)	Communion (− 3;3)	Luck (− 3;3)
HA/LC	1.56	− 1.09	0.29
LA/HC	− 1.88	0.29	0.03
HA/HC	2.50	2.06	0.26
LA/LC	− 2.00	− 1.62	− 0.12
Good luck	− 0.21	0.38	1.32
Bad luck	− 0.53	0.18	− 2.29

Note: Descriptions same as in Table 1. HA = High Agency, LA = Low Agency, HC = High Communion, LC = Low Communion

### Discussion

Results of Experiment 2 together with Experiment 1 demonstrated that participants do not exhibit a positive illusion of control by proxy, neither for proxies with high agency nor for ones with a high luck. Results show, however, that there was a significant negative illusion of control by proxy, manifested by an avoidance of proxies carrying negative qualities – low agency or low luck.

These results are inconsistent with the findings by Wohl and Enzle (2009), however there may be an easy explanation as to why their effect was not replicated. A very important difference between the study by Wohl and Enzle and our study was the description of the lucky proxy. As mentioned earlier, Wohl and Enzle used a backstory about touching a lucky coin which made the proxy ‘unable to lose’, effectively a lottery demigod. Our description of the lucky proxies was much more mundane, stating that the lucky proxy won small to moderate amounts of money several times. The aim of this description was to avoid a possible scenario in which participants would let the lucky proxy choose the lottery ticket (or number) out of pure curiosity, to see whether the proxy is indeed so lucky. It seems that results by Wohl and Enzle may very well be explained by such a mechanism.

To further investigate the issue of how the descriptions of proxies influence participants’ preferences, and in an attempt to obtain a positive illusion of control by proxy, we decided to strengthen the descriptions of proxies in Experiment 3. Our hypothesis was that proxies with a very high agency and very high luck would be preferred over RNG.

## Experiment 3

The aim of Experiment 3 was to test whether the lack of ‘positive’ illusion of control by proxy in the previous two experiments was caused by the descriptions of agency, communion and luck being perceived as too mundane and ‘normal’ by the participants, i.e. a failure to elicit enough belief in the proxies’ positive traits to warrant choosing them over own control. We therefore decided to make the descriptions of positive agency, communion and luck stronger, but still possible in the real world. In case of agency and communion, we decided to substitute situational descriptions of particular acts (which are by definition one-time and do not necessarily reflect a person’s general traits) for direct descriptions of traits. For example, a proxy with high agency would be described in Experiments 1 and 2 with the sentence ‘He knew the current stock market trends’, which only reflects one particular situation. Instead, in the current experiment a high agency proxy would be described as ‘People say that he is competent and scrupulous’ – a general description of the proxy’s recurring characteristics. We hypothesized that this change would increase the perceived reliability of the proxy as a high-agency person, therefore prompting a stronger preference of proxy over own control.

In case of positive luck, we wanted to make the proxies’ lucky feats more impressive, yet still more believable than those presented by Wohl and Enzle (2009). Again, we hypothesized that this modification would prompt a stronger preference of the lucky proxies over own control than observed in the previous experiments.

### Participants

Seventy-four students (60 females, 13 males, one person did not indicate their gender) aged 18–28 years (M = 20.83, SD = 1.83) took part in the experiment. They were recruited on campus, and didn’t receive payment for participation (the recruitment procedure was the same as in the Experiment 1 and 2). Informed consent was obtained from all participants. Sample size was similar to the one used in Experiment 2.

### Materials and Procedure

The study was run using the same procedure as in Experiment 2, which included three choices between one proxy and the random number generator. Since we wanted to investigate the positive cases, in which we did not obtain an illusion of control by proxy, we introduced three types of proxies: (1) High Agency (with no information about other traits), (2) High Communion (with no information about other traits), and (3) High Luck (same). The order of the proxies presented was randomized. There were two counterbalancing sets differing in the descriptions of proxies, each given to 50% of the participants. As regards the High Luck proxy, we decided to implement two different descriptions of luck: one lucky proxy had a single, but very impressive lucky feat (winning the grand prize at a national lottery), while the other lucky proxy had smaller, but recurring lucky feats (i.e. very often winning small to moderate amounts of money in lotteries). This differentiation was included for three reasons. Firstly, we wanted to separately cover the two aspects of expected utility maximization (Bernoulli, 1738, as cited in Kahneman and Tversky, 2013; von Neumann and Morgenstern, 1944) – probability and outcome. Secondly, it is possible that a single lucky feat would prompt thinking in terms of the gambler’s fallacy (Mowrer, Rayman & Bliss, 1940; Huber, Kirchler & Stoeckl, 2010). Participants could expect the person who won the grand prize in a lottery to ‘run out’ of luck and be useless as a lucky control proxy – which would not be true for the person presenting regular small lucky feats. Finally, the description by Wohl and Enzle was akin to our lucky proxy with small, but regular feats, so we wanted to check whether the potential illusion of control by proxy would generalize to another type of very lucky proxy.

### Results

**Fig. 5:**
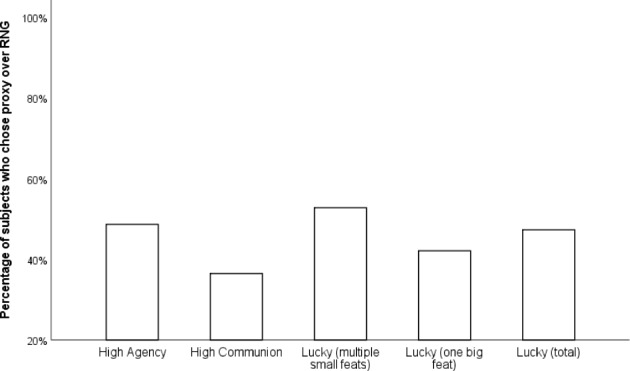
Percentages of participants who chose proxy over RNG (Experiment 3)

Participants’ choices were compared to 50% random chance by means of chi-square tests. It turned out that, contrary to the hypothesis, participants still did not have significant preferences between the High Agency proxy and RNG (36 vs. 38, χ^2^(1,N = 74) = 0.054, Cramer’s V = 0.03). However, there was a moderate preference of RNG over a High Communion proxy (47 RNG vs. 27 Proxy; χ^2^(1,N = 74) = 5.405, Cramer’s V = 0.27). There were negligible preferences between a High Luck proxy and RNG (35 vs. 39, χ^2^(1,N = 74) = 0.216, Cramer’s V = 0.05). Separating the lucky proxies into the two sub-categories revealed negligible preferences between the lucky proxy with multiple small feats and RNG (19 vs. 17, χ^2^(1,N = 36) = 0.111, Cramer’s V = 0.06) but showed a weak preference of RNG over the lucky proxy with a single impressive (16 vs. 22, χ^2^(1,N = 38) = 0.947, Cramer’s V = 0.16). Results are presented in Fig. 5.

Figure 5. Percentages of participants who chose proxy over RNG (Experiment 3).

Post-experimental questionnaires showed that the perceived qualities of proxies were consistent with their descriptions apart from one case: the High Communion proxy was also perceived as having moderately positive agency. Perceived qualities of proxies are presented in Table 3.

**Table 3:** Examples of descriptions of proxies and their perceived qualities (Experiment 3)

Type	Description	Agency (− 3;3)	Communion (− 3;3)	Luck (− 3;3)
HA (1)	*People say he’s effective and has a broad knowledge*	1.94	0.91	0.63
HA (2)	*People say he’s competent and scrupulous*			
HC (1)	*People say he’s honest and moral*	1.12	1.76	0.38
HC (2)	*People say he’s truthful and loyal*			
Good luck (1)	*He has played various lotteries for several years. He very often wins small prizes.*	0.39	0.42	1.91
Good Luck (2)	Last year he won the grand prize* in the national lottery [Comment: *est. value of prize between $250k-$10 M]			

Note: HA = High Agency, HC = High Communion

### Discussion

Results of Experiment 3, while mostly statistically insignificant, are consistent with the results of the two previous experiments. We were unable to obtain an illusion of control by proxy in any of the cases. We did obtain another instance of the ‘negative’ illusion of control by proxy for the High Communion proxy, which is consistent with previous results obtained in Experiment 2 (preference of RNG over a low agency – high communion proxy) – the perceived high communion of an agent is not a reason for participants to give up control to such an agent with the absence of information about their agency or luck. This result is therefore not supportive of the ‘just world’ mechanism. The fact that we did not obtain a significant preference for the lucky proxy over RNG seems to indicate that the results by Wohl and Enzle (2009) do not replicate in the modified procedure, at least not in the Polish cultural context. It could be explained by results of studies showing that young Poles (who were the participants in our study) do not believe in the ‘just world’ in terms of Lerner theory (Skrzypinska, 2003). It is also possible that Wohl and Enzle’s results were caused by the participants’ desire to test the proxy’s unbelievable luck for themselves, therefore showing a case for cognitive needs, rather than illusion of control.

## General Discussion

The three experiments presented in this paper failed to obtain the illusion of control by proxy, as presented by Wohl and Enzle (2009). Participants s were largely indifferent when choosing between RNG and proxies with high agency and good luck. High communion alone, or coupled with low agency, prompted participants to avoid such a proxy in favor of RNG, as did bad luck. These two last effects indicate that participants indeed consider the traits of a proxy as relevant to the outcome of a random chance game, which is consistent with the definition of illusion of control. Moreover, the traits taken into consideration include not only personal luck, but also agency. Participants avoiding a proxy who has low agency (i.e. an incompetent financial advisor) in the context of providing numbers for a random chance game with an unknown outcome, shows a violation of rational expectations. A rational agent should not believe that a financial advisor (good or bad, competent or incompetent, or carrying any other traits) would be better or worse at selecting winning lottery numbers than a random number generator – especially since the winning numbers were yet to be chosen, so no insider information was possible.

We did not obtain the ‘positive’ illusion of control by proxy, which would manifest itself in participants choosing proxies with good qualities over themselves. This is all the more surprising, since we provided participants with a choice of proxy vs. RNG, which was hypothesized to reduce the influence of their own illusion of control as compared to the choice of proxy vs. own choice (as in Wohl and Enzle, 2009). Therefore the result does not seem attributable to the tradeoff between own illusion of control and illusion of control by proxy, but more likely to population characteristics (preventative, rather than promotional illusory control in the Polish sample) and/or the fact that the descriptions of proxies did not allow participants to ‘call bluff’ on their luck or other abilities. These possibilities were not directly tested in the present study, and should be investigated in future research.

Most importantly, all three experiments provide consistent evidence for a ‘negative’ or preventative illusion of control for proxies carrying undesired qualities – low agency, bad luck, or no information about agency or luck. This result adds to the research by Wohl and Enzle (2009) showing that an illusion of control by proxy can occur for proxies with negative qualities.

The main issue with the presented research is that we failed to replicate the illusion of control by proxy as presented by Wohl and Enzle. There is a need for a direct replication of their research in another cultural context to rule out that their results were only specific to Canada. More importantly, it seems that the illusion of control by proxy is sensitive to procedural changes which are unclear at the moment. Our results may indicate that the illusion of control by proxy is actually not what Wohl and Enzle (2009) described. In fact, illusion of control by proxy may have been an experimental artifact, caused by other mechanisms. In order to speculate on these mechanisms, let us discuss the differences between the research by Wohl and Enzle (2009), where the illusion of control by proxy was observed, and our research, where it was absent. First of all, as mentioned earlier, the manipulation by Wohl and Enzle may have been too strong, causing participants to choose the lucky proxy just out of curiosity, to see whether they are indeed so lucky. In the same vein, the lucky proxy spending a lot of time to tell their story and show their lucky past may have caused a degree of social pressure – it may have been considered rude not to ask the proxy to use their luck, which they talked about so much. In other words, using the proxy may have been a demonstration of conformity (normative or informative), rather than illusion of control. Finally, the proxy’s story of luck was a focal point in Wohl and Enzle’s procedure, which may have simply caused complacency with experimental demand characteristics (Orne, 1962).

On the other hand, the random number generator may have been treated by some participants as an extension of own illusory control – the only choice by which they could have exercised their own personal luck. This, however, would have been even more visible in the original research by Wohl and Enzle, since they allowed participants to choose the lottery ticket themselves, which is a more directly controllable situation than using RNG.

Finally, there is the issue of justifying one’s choices. We know from research that a clear justification of a choice influences preferences (e.g. Tversky & Shafir’s, 1992 disjunction effect). Let us assume that participants do not believe in luck, and do not attempt to exert illusory control, and hence are indifferent between proxy and RNG. When faced with the choice between proxy and RNG which they have to make, they therefore ask themselves: *Why should I choose the proxy?* In Wohl Enzle’s (2009) control conditions, where the participant was neither lucky nor unlucky, there really was no justification to ask them to choose the lottery tickets for us. However, when the proxy presented their luck, it provided sufficient justification, even if a given participant did not believe the proxy would actually be lucky. Similarly in our research, when faced with the choice between RNG and negative proxies (low agency, low communion, unlucky), there was enough justification for participants to decide that they do not want such a proxy to help them, even if they did not believe it would actually cause any negative results.

The indifference between most of the positive proxies and RNG in our research would then be explained by participants asking themselves “*why should I choose proxy over RNG?”* and generally not finding enough justification for either option. This pattern of results may also reflect the negativity effect. Many previous studies showed that negative information weighs more than positive - negative attributes get more attention, are better stored in memory and have a greater impact on people than positive evaluations (for a review, see Baumeister et al., 2001). Following that, it is possible that in our research positive attributes were not enough to choose a proxy but negative attributes were enough not to choose him.

While the above issues cast a shadow of doubt over the illusion of control by proxy, a series of direct replications of their experiments is needed to obtain potential proof that the effect does not exist at all. However, if we assume that the effect exists, the future research is also needed to further investigate the conditions under which the illusion of control occurs and does not occur. For instance, it could be worthwhile to check how the respondents evaluate their own luck in random chance games or their own financial knowledge. It is possible that self- comparison may influence the decision about choosing the proxy - people are only willing to pass on control if they consider others to be more competent or lucky than they are.

The main goal of the present study was to demonstrate that illusion of control goes beyond the domains of ‘personal luck’ and ‘skill elements of a situation’, and that various traits and qualities of agents are considered when deciding whether to ask them for ‘support’ in games of chance. Our results support this assumption, even though we only obtained significant results for negative qualities (who not to ask for help). This indicates that attempts at controlling random chance events may be based not only on perceived ‘skill’ (Langer, 1975) and exercising ‘personal luck’ (Wohl & Enzle, 2003, 2004), but may be a more general tendency to consider any factors differentiating the various options, with the general assumption that a better option (a more attractive proxy, RNG over an unattractive proxy, etc.) is more favorable, regardless of the domain. We desire control over events of random chance, and refuse to accept ambiguity, so we may be willing to use any qualities which differentiate between options, even as ‘absurd’ as being an incompetent or immoral financial advisor. As such, factors influencing the illusion of control (and illusion of control by proxy in particular) may be much more general than previously assumed.

### Limitations

The study has some limitations, which need to be addressed in subsequent research to increase the generalizability of the results. First, the sample consisted of students and young people, who should not be considered representative of the general population, let alone of occasional gamblers or problem gamblers – behavior of gamblers may deviate from what our results showed. Moreover, research is needed to capture the mechanisms of why and when the illusion of control by proxy occurs and doesn’t occur – a major problem with this research was that we were unable to replicate Wohl and Enzle’s, 2009 results. At the very least, participants’ perceived personal luck and illusion of control should be measured, as it counteracts the need to ask the proxy for their personal luck. Another limitation may stem from the use of a computer-assisted procedure, which may reduce the perceived realism of the gambles – a replication using casino equipment and direct personal contact with the proxies presenting high or low luck/agency/communion may yield different results. Finally, replications in contexts of various types of lotteries (various chances of winning, various perceived skill elements) are needed.

### Implications

The main implication of the findings is consistent with the notion that when faced with risk or uncertainty (in gambling or elsewhere), we seek ways to reduce this uncertainty, which is the motivation behind many cognitive biases (cf. Kahneman, 2011). Gamblers are especially exposed to risk due to the mere nature of gambling. The findings indicate that people believe certain characteristics of other people influence their chances of winning (specifically, that low agency and/or communion reduces these chances), so there may be more to the illusion of control and the illusion of control by proxy, than just referring to one’s skill or luck. These beliefs, in turn, may influence gambling behavior, serving as a substitute of rational probability analysis and expectations. Research is therefore needed to investigate other general elements of social perception and self-perception which may influence gambling behavior.

## Footnotes

1 Equivalent to USD 2.85, perceived value according to the Big Mac Index = USD 5.07.

## References

[CR1] Abele, A. E., & Bruckmüller, S. (2011). The bigger one of the “Big Two”? Preferential processing of communal information. *Journal of Experimental Social Psychology*, *47*(5), 935–948. 10.1016/j.jesp.2011.03.028

[CR2] Abele, A. E., & Wojciszke, B. (2007). Agency and communion from the perspective of self versus others. *Journal of Personality and Social Psychology*, *93*(5), 751–763. 17983298 10.1037/0022-3514.93.5.751

[CR3] Abele, A. E., & Wojciszke, B. (2014). Communal and agentic content in social cognition: A dual perspective model. *Advances in Experimental Social Psychology*, *50*, 195–255. 10.1016/B978-0-12-800284-1.00004-7

[CR4] Bandura, A. (2001). Social cognitive theory: An agentic perspective. *Annual Review of Psychology*, *52*(1), 1–26. 11148297 10.1146/annurev.psych.52.1.1

[CR5] Baumeister, R. F., Bratslavsky, E., Finkenauer, C., & Vohs, K. D. (2001). Bad is stronger than good. *Review of General Psychology*, *5*, 323–370. 10.1037/1089-2680.5.4.323

[CR6] Bruner, J. S., Goodnow, J. J., & Austin, G. A. (1956). *A study of thinking*. New York: Wiley.

[CR8] Callan, S., Harvey, & Dawtry (2014). Immanent justice reasoning. Theory, research and current directions. *Advances in Experimental Social Psychology*, *49*, 106–161.

[CR7] Callan, E., & Nicole (2006). The belief in a Just World and Immanent Justice reasoning in adults. *Personality and Social Psychology Bulletin*, *42*, 1646–1658. 10.1177/0146167206292236 17122177

[CR9] Darke, P. R., & Freedman, J. L. (1997). The belief in good luck scale. *Journal of research in personality*, *31*(4), 486–511. 10.1006/jrpe.1997.2197

[CR10] Ejova, A., Delfabbro, P. H., & Navarro, D. J. (2015). Erroneous gambling-related beliefs as illusions of primary and secondary control: A confirmatory factor analysis. *Journal of Gambling Studies*, *31*(1), 133–160. 23861012 10.1007/s10899-013-9402-9

[CR14] Goodie, A. S., Fortune, E. E., & Shotwell, J. J. (2019). Cognitive distortions in disordered gambling. *Gambling disorder* (pp. 49–71). Cham: Springer.

[CR12] Griffiths, M. (1999). Gambling technologies: Prospects for problem gambling. *Journal of Gambling Studies*, *15*(3), 265. 12766464 10.1023/A:1023053630588

[CR13] Griffiths, M. D., & Bingham, D. (2005). A study of superstitious beliefs among bingo players. *Journal of Gambling Issues (JGI)*, (13).

[CR15] Henslin, J. M. (1967). Craps and magic. *American Journal of Sociology*, *73*(3), 316–330. 10.1086/224479

[CR16] Higgins, E. T. (1997). Beyond pleasure and pain. *American Psychologist*, *52*(12), 1280. 9414606 10.1037/0003-066X.52.12.1280

[CR17] Higgins, E. T. (1998). Promotion and prevention: Regulatory focus as a motivational principle. *Advances in experimental social psychology* (30 vol., pp. 1–46). Academic Press.

[CR18] Huber, J., Kirchler, M., & Stöckl, T. (2010). The hot hand belief and the gambler’s fallacy in investment decisions under risk. *Theory and Decision*, *68*(4), 445–462. 10.1007/s11238-008-9106-2

[CR19] Joukhador, J., Maccallum, F., & Blaszczynski, A. (2003). Differences in cognitive distortions between problem and social gamblers. *Psychological Reports*, *92*(3_suppl), 1203–1214. 12931940 10.2466/pr0.2003.92.3c.1203

[CR20] Kahneman, D. (2011). *Thinking, fast and slow*. Allen Lane.

[CR21] Kahneman, D., & Tversky, A. (1979). Prospect theory: An analysis of decision under risk. *Econometrica*, *47*(2), 363–391. 10.2307/1914185

[CR22] Kahneman, D., & Tversky, A. (2013). Choices, values, and frames. In *Handbook of the Fundamentals of Financial Decision Making: Part I* (pp. 269–278).

[CR23] Keren, G., & Wagenaar, W. A. (1988). Chance and skill in gambling: A search for distinctive features. *Social Behaviour*.

[CR24] Langens, T. A. (2007). Regulatory focus and illusions of control. *Personality and Social Psychology Bulletin*, *33*(2), 226–237. 17259583 10.1177/0146167206293494

[CR25] Langer, E. J. (1975). The illusion of control. *Journal of Personality and Social Psychology*, *32*(2), 311. 10.1037/0022-3514.32.2.311

[CR26] Langer, E. J. (1983). *The psychology of control*. Beverly Hills: Sage Publications.

[CR27] Lee, D. K. (2016). Alternatives to P value: Confidence interval and effect size. *Korean Journal of Anesthesiology*, *69*(6), 555. 27924194 10.4097/kjae.2016.69.6.555 PMC5133225

[CR28] Lichtenstein, S., & Slovic, P. (1971). Reversals of preference between bids and choices in gambling decisions. *Journal of Experimental Psychology*, *89*(1), 46. 10.1037/h0031207

[CR29] Louviere, J. J., Hensher, D. A., & Swait, J. D. (2000). *Stated choice methods: Analysis and applications*. Cambridge University Press.

[CR30] Moore, S. M., & Ohtsuka, K. (1998). *Control over gambling: Solution or problem?* (Doctoral dissertation, National Association for Gambling Studies).

[CR31] Mowrer, O. H., Rayman, N. N., & Bliss, E. L. (1940). Preparatory set (expectancy)—an experimental demonstration of its’ central’locus. *Journal of Experimental Psychology*, *26*(4), 357. 10.1037/h0058172

[CR32] Nemeroff, C., & Rozin, P. (2000). The makings of the magical mind: The nature and function of sympathetic magical thinking. In K. S. Rosengren, C. N. Johnson, & P. L. Harris (Eds.), *Imagining the impossible: Magical, scientific, and religious thinking in children* (pp. 1–34). New York, NY, US: Cambridge University Press.

[CR33] Nickerson, R. S. (1998). Confirmation bias: A ubiquitous phenomenon in many guises. *Review of General Psychology*, *2*(2), 175–220. 10.1037/1089-2680.2.2.175

[CR34] Orne, M. T. (1962). On the social psychology of the psychological experiment: With particular reference to demand characteristics and their implications. *American Psychologist*, *17*(11), 776. 10.1037/h0043424

[CR35] Peeters, G. (2001). In search for a social – behavioral approach – avoidance dimension associated with evaluative trait meanings. *Psychologica Belgica*, *41*(4), 187–203. 10.5334/pb.980

[CR36] Peirce, J. W., Gray, J. R., Simpson, S., MacAskill, M. R., Höchenberger, R., Sogo, H., Kastman, E., & Lindeløv, J. (2019). PsychoPy2: Experiments in behavior made easy. *Behavior Research Methods*. 10.3758/s13428-018-01193-y 30734206 10.3758/s13428-018-01193-y PMC6420413

[CR37] Raylu, N., & Oei, T. P. (2004). The Gambling related Cognitions Scale (GRCS): Development, confirmatory factor validation and psychometric properties. *Addiction*, *99*(6), 757–769. 15139874 10.1111/j.1360-0443.2004.00753.x

[CR38] Skrzypinska, K. (2003). Dlaczego młodzi Polacy nie wierzą w świat sprawiedliwy, ale wierzą w człowieka [Why young Poles do not believe in a just world but believe in human nature]. In B. Wojciszke, M.Plopa (Ed.), *Osobowość i procesy psychiczne [Personality and cognitive processes]* (pp. 5–14). Oficyna Wydawnicza Impuls.

[CR39] Steenbergh, T. A., Meyers, A. W., May, R. K., & Whelan, J. P. (2002). Development and validation of the gamblers’ beliefs Questionnaire. *Psychology of Addictive Behaviors*, *16*(2), 143. 12079253 10.1037/0893-164X.16.2.143

[CR40] Toneatto, T. (1999). Cognitive psychopathology of problem gambling. *Substance Use & Misuse*, *34*(11), 1593–1604. 10468110 10.3109/10826089909039417

[CR41] Torgler, B. (2007). Determinants of superstition. *The Journal of Socio-Economics*, *36*(5), 713–733.Tversky, A., & Shafir, E. (1992). The disjunction effect in choice under uncertainty. *Psychological Science, 3* (5), 305–310.

[CR42] von Neumann, J., & Morgenstern, O. (1944). *Theory of Games and Economic Behavior*, Second edition, 1947; third edition, 1953. Princeton, New Jersey: Princeton University Press.

[CR43] Wiseman, R., & Watt, C. (2004). Measuring superstitious belief: Why lucky charms matter. *Personality and Individual Differences*, *37*(8), 1533–1541. 10.1016/j.paid.2004.02.009

[CR44] Wohl, M. J., & Enzle, M. E. (2002). The deployment of personal luck: Sympathetic magic and illusory control in games of pure chance. *Personality and Social Psychology Bulletin*, *28*(10), 1388–1397. 10.1177/014616702236870

[CR45] Wohl, M. J., & Enzle, M. E. (2003). The effects of near wins and near losses on self-perceived personal luck and subsequent gambling behavior. *Journal of Experimental Social Psychology*, *39*(2), 184–191. 10.1016/S0022-1031(02)00525-5

[CR46] Wohl, M. J., & Enzle, M. E. (2009). Illusion of control by proxy: Placing one’s fate in the hands of another. *British Journal of Social Psychology*, *48*(1), 183–200. 18034916 10.1348/014466607X258696

